# A combined diagnostic model including middle rectal artery visualization for predicting lateral lymph node metastasis in rectal cancer

**DOI:** 10.3389/fphys.2024.1444897

**Published:** 2025-01-07

**Authors:** Ning Wang, Yiping Li, Kun Lu, Kaikai Wei, Shize Jia, Shuhong Fan, Donglin Ren, Yuanji Fu, Zhimin Liu

**Affiliations:** ^1^ Department of Radiology, The Sixth Affiliated Hospital, Sun Yat-sen University, Guangzhou, China; ^2^ Department of General Surgery (Coloproctology), The Sixth Affiliated Hospital, Sun Yat-sen University, Guangzhou, China; ^3^ Guangdong Provincial Key Laboratory of Colorectal and Pelvic Floor Diseases, The Sixth Affiliated Hospital, Sun Yat-sen University, Guangzhou, China; ^4^ Biomedical Innovation Center, The Sixth Affiliated Hospital, Sun Yat-sen University, Guangzhou, China; ^5^ Department of Anesthesia, The Sixth Affiliated Hospital, Sun Yat-sen University, Guangzhou, China; ^6^ School of Public Health, Sun Yat-sen University, Guangzhou, China

**Keywords:** rectal cancer, middle rectal artery, lateral lymph node, computed tomography, diagnostic test

## Abstract

**Purpose:**

This study attempted to establish a combined diagnostic model encompassing visualization of the middle rectal artery (MRA) and other imaging features to improve the diagnostic efficiency of lateral lymph node (LLN) metastasis, which is crucial for clinical decision-making in rectal cancer.

**Method:**

One hundred eleven patients receiving bilateral or unilateral lymph node dissection were enrolled, and 140 cases of LLN status on a certain unilateral pelvic sidewall were selected. Enhanced computed tomography (CT) was used to determine whether MRA was visible. Multivariable regression was used to establish a diagnostic model combining MRA visualization with other imaging features to predict LLN metastasis. Receiver operating characteristic (ROC) curve and area under the ROC curve (AUC) were used to test the diagnostic efficacy for LLN metastasis. Ten-fold cross-validation was completed to internally validate the diagnostic model.

**Results:**

Of the 140 LLNs harvested from 111 patients, 76 were positive and 64 were negative for metastases, respectively. The diagnostic model combining the MRA visualization and lymph node short diameter showed a greater efficiency than a single scale (AUC = 0.945, 95% confidence interval = 0.893–0.976, *P* < 0.001). The mean cross-validated AUC was 0.869 (95% confidence interval = 0.835–0.903).

**Conclusion:**

Our results establish a combined diagnostic model with the help of MRA visualization to yield a high diagnostic efficiency of LLN metastasis in rectal cancer.

## Introduction

Lateral lymph node (LLN) metastasis occurs occasionally with a prevalence of about 8%–49% in lower rectal cancer (Ueno et al., 2005; Ogawa et al., 2021; Akiyoshi et al., 2012; Yano and Moran, 2008). LLN metastasis in rectal cancer is associated with increased local recurrence and poorer survival (Ueno et al., 2005; Akiyoshi et al., 2012; Schaap et al., 2021). Until now, the clinical management of LLN metastasis has differed between the East and the West. Medical institutions in Japan tend to treat LLN metastasis with lateral lymph node dissection (LLND) and total mesorectal excision, while preoperative neoadjuvant therapy with total mesorectal excision, in which LLNs are not resected automatically, is completed more commonly than LLND in Western countries (Ogawa et al., 2021; Akiyoshi et al., 2012; Williamson et al., 2020). Imaging diagnosis of LLN metastasis is crucial for the clinical decision-making in lower rectal cancer (Yano and Moran, 2008).

Lower rectal cancer is more inclined to lateral lymphatic drainage, possibly along the middle rectal artery (MRA), to the internal iliac vessel (Yoo et al., 2023). The MRA originates from the internal iliac artery; connects the rectum with the lateral pelvic wall; and can be divided into anterolateral, lateral, and posterolateral types (Kiyomatsu et al., 2017). Recently, the MRA was considered a predicter of LLN metastasis (Iwasa et al., 2021). The diameter of the MRA fluctuates from 0.5 to 3.5 mm (Heinze et al., 2023), so it can be clearly seen on thin-slice enhanced computed tomography (CT) images. In addition, given its high popularity, wide field of view, thin layers and various reconstruction methods, CT may be more conducive than magnetic resonance imaging to visualization of the MRA and small lymph nodes.

At present, the imaging diagnosis of LLN metastasis mainly depends on lymph node size (Ogawa et al., 2021; Amano et al., 2020; Ogawa et al., 2016a). Studies on the correlation between the MRA on CT images and LLN metastasis in rectal cancer are lacking. Therefore, the present study attempted to explore the relationship between the MRA and LLN metastasis on CT images, whether MRA visualization on a certain lateral side can predict LLN metastasis on the corresponding lateral side, and whether MRA visualization on CT images combined with lymph node short diameter can improve the diagnosis of LLN metastasis.

## Materials and methods

### Patients

**FIGURE 1:**
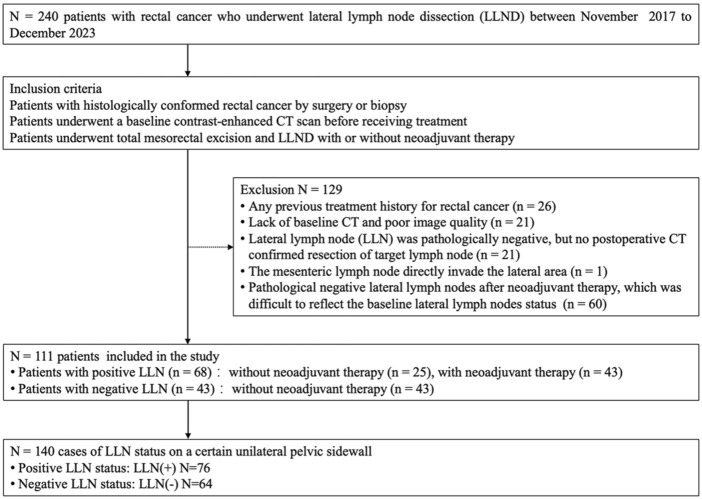
Flowchart of participants of the study (LLND = lateral lymph node dissection, LLN = lateral lymph node).

This retrospective study was approved by the institutional ethics committee, and the requirement for written informed consent was waived. A total of 240 patients with histologically confirmed rectal cancer underwent LLND between November 2017 and December 2023 at our institution. Among these patients, 26 were excluded because of a history of total mesorectal excision, 21 were excluded due to lack of baseline CT imaging or poor image quality, 21 were excluded due to an absence of postoperative CT confirmation of resection of the target lymph node, and one was excluded because the lateral metastasis was directly invaded by the mesenteric lymph node. An additional 60 patients with negative LLNs after neoadjuvant therapy were also excluded due to difficulty reflecting the baseline LLNs status. Finally, 111 patients were enrolled, with some receiving bilateral LLND and the rest receiving unilateral LLND. There were 140 cases of LLN status on a certain unilateral pelvic sidewall, of which 76 metastatic LLNs were divided into the LLN(+) group and 64 benign LLNs were divided into the LLN(−) group (Figure 1). The clinical characteristics, primary tumor, and regional lymph node pathological features of patients with rectal cancer were recorded by consulting medical records.

### MRA visualization on CT

The MRA originates from the anterior division of the internal iliac artery and branches either from the internal pudendal artery, the inferior gluteal artery, the gluteal pudendal trunk, or directly from the internal iliac artery. The MRA may have one or more branches that flow from the pelvic sidewall, penetrate through the mesorectal fascia, and communicate with the arterial system in the mesorectum or directly run into the rectum below the peritoneal reflection and above the levator anal muscle. The MRA may be accompanied by several venous and lymphatic vessels (Kiyomatsu et al., 2017; Heinze et al., 2023).

**FIGURE 2:**
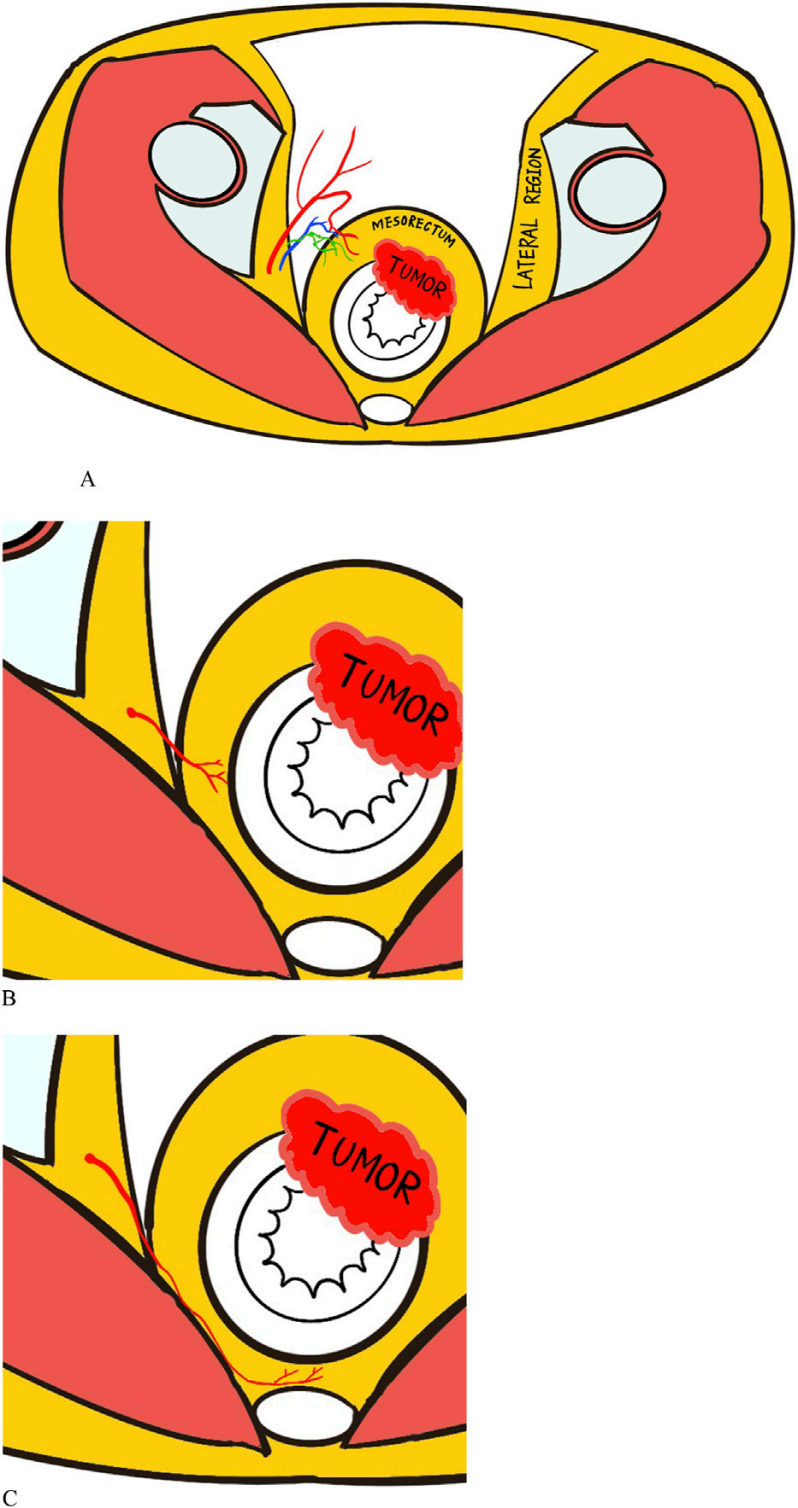
Illustrations of the anatomy of middle rectal artery (MRA) with three types: **(A)** Anterolateral type. **(B)** Lateral type. **(C)** Posterolateral type. The red squiggly lines connecting lateral region and rectum represent the MRA, while the blue and green lines represent the accompanying venous and lymphatic vessels.

The MRA is divided into three types according to the vessel path, including anterolateral, lateral, and posterolateral types (Figure 2). Anterolateral MRAs penetrate the mesorectal fascia through the anterolateral or anterior side and end up into the anterior wall of the rectum, which usually has a long common trunk with the prostatic artery or the inferior vesical artery. The lateral type of MRA is one of the major independent branches from the anterior division of the internal iliac artery that perforates laterally through the mesorectal fascia into the rectum. Finally, the posterolateral type of MRA travels a long way backward between the mesorectal fascia and the anterior sacral fascia before eventually entering the posterior wall of the rectum. The different individual types of MRA can be identified on enhanced CT images by tracing their anatomical structure and relationships.

In this retrospective study, we employed the Aquilion ONE TSX-301A 640-slice CT scanner (Toshiba, Tokyo, Japan) with a thickness of 1 mm and the Optima CT 660 scanner (GE Medical Systems, Chicago, IL, United States) with a thickness of 1.25 mm. Preoperative and postoperative CT imaging was performed using a gastrointestinal protocol with arterial, venous, delayed as the three-phases using intravenous nonionic iodinated contrast agents (3.0 mL/s).

**FIGURE 3:**
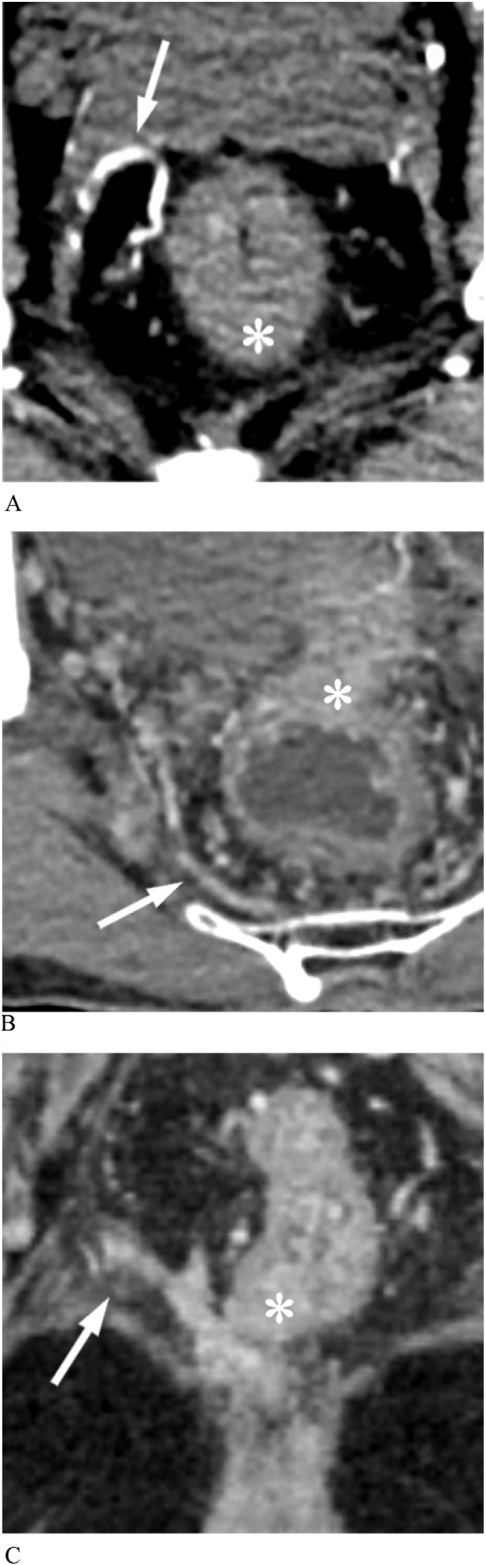
Middle rectal arteries (MRAs) display on enhanced CT images. **(A)** Arterial phase image shows that an anterolateral MRA (arrow) penetrates the mesorectal fascia into the rectal tumor (asterisk). **(B)** Venous phase image shows a posterolateral MRA (arrow) communicating pelvic plexus with the rectal tumor (asterisk). **(C)** The rectal cancer (asterisk) has a protrusion of stripes connected to the pelvic vessel branch, representing MRA (arrow).

The MRA was visible if one or more branches of the internal iliac vessels connected the pelvic sidewall and the mesorectum on enhanced CT images, with specific imaging details as follows (Figure 3):

A. Enhanced arterial-phase images clearly showed one or more arteries originating from the internal iliac artery and entering the rectum at the tumor level.
B. Enhanced venous-phase images clearly showed one or more vessels connecting the pelvic sidewall with the rectum, which may be obscure in the arterial phase.
C. Enhanced images showed that the lateral vessels originating from the internal iliac vessels were not clearly demarcated from the rectal tumor invading the mesorectal fascia.

Two radiologists with 5 and 8 years of experience, respectively, in diagnosing gastrointestinal diseases evaluated the MRA visualization on the corresponding lateral side in each case. The readers were blinded to the histological results of the rectal cancer and the LLN status. A consensus was reached by discussing the visualization of MRA in each case where there was a disagreement between the two readers. All images were evaluated using the PACS software (Vue RIS 3.2.0003.0; Carestream, Geneva, Switzerland).

### Image features

The most suspected metastatic LLN was identified as the target lymph node on baseline CT images. The target LLNs usually had the largest short diameter, irregular margins, and heterogeneous density. The removal of the target LLN must be confirmed by comparing postoperative and baseline CT images if the pathology of the LLN is negative. The anatomical region of the LLNs can be divided into the internal iliac, obturator, and external iliac compartments (Sluckin et al., 2022; Ogura et al., 2019). The short diameter and region of the target LLN were recorded in each case. Other imaging features, including clinical T staging, mesorectal lymph node status, extramural vascular invasion (EMVI) (Jhaveri et al., 2016), and distance from the inferior margin of the tumor to the anal margin were evaluated by baseline CT and MRI (Table 1).

**TABLE 1:** Clinicopathological and imaging features of patients with positive and negative LLN^a^.

Features	Patients with positive LLN (n = 68)	Patients with negative LLN (n = 43)	P value
Age (years)^b^	54 ± 13.5	59 ± 10.9	0.019
Gender^c^			0.062
Male	37 (54)	31 (72)	
Female	31 (46)	12 (28)	
Anal distance (mm)^d^	42 (29–53)	57 (40–70)	0.001
Clinical T stage			0.010
1–2	1 (1)	7 (16)	
3–4	67 (99)	36 (84)	
Mesenteric lymph node on images			0.003
Positive	57 (84)	25 (58)	
Negative	11 (16)	18 (42)	
EMVI^a^ on images			<0.001
Positive	40 (59)	6 (14)	
Negative	28 (41)	40 (87)	
Pathological type			0.404
Adenocarcinoma	58 (85)	39 (91)	
Others	10 (15)	4 (9)	
Pathological grade^e^			0.050
Highly-Moderately differentiated	45 (78)	37 (93)	
Poorly differentiated	13 (22)	3 (7)	
Pathological T stage^f^			0.018
1–2	4 (16)	19 (44)	
3–4	21 (84)	24 (56)	
Pathological mesenteric lymph node status^f^			<0.001
Positive	21 (84)	12 (28)	
Negative	4 (16)	31 (72)	
Intravascular invasion^f^			0.252
Positive	11 (44)	13 (30)	
Negative	14 (56)	30 (70)	
Nerve invasion^f^			0.017
Positive	9 (36)	5 (12)	
Negative	16 (64)	38 (88)	

Note:

^a^ Abbreviations: LLN, lateral lymph node; EMVI, extramural vascular invasion.

^b^ Normally distributed variable was shown as Mean ± standard deviation.

^c^ Categorical variable was shown as n (%).

^d^ Nonnormally distributed variable was shown as the Median (interquartile range).

^e^ Unknown cases were excluded.

^f^ Only patients without neoadjuvant chemoradiotherapy were included.

### Statistical analysis

According to previous literature (Ogawa et al., 2016a; Hiyoshi et al., 2020; Ogawa et al., 2017; Abe et al., 2022), factors that generally affect LLN metastasis include the short diameter of the lymph node, histological type, T stage, status of the mesenteric lymph node, EMVI, and distance from the anal margin. The main predictor explored in this study was the MRA visualization. Therefore, the regression equation for predicting LLN metastasis was estimated to include seven variables. Based on the rule of examining one predictor in at least five events and assuming a 40% probability of negative patients without LLN metastasis, given that the most cases are therapeutic LLN resection, at least 88 patients should be included.

The continuous-variable normality test was performed using the Shapiro-Wilk normality test. Normally distributed variables are shown as the mean ± standard deviation, whereas non-normally distributed variables are presented as the median and interquartile range (IQR). Agreement between the two radiologists was evaluated with Cohen’s kappa statistics. The comparison of continuous quantitative variables between the negative and positive patients or LLN groups was performed using the T-test or Mann-Whitney U non-parametric test according to the results of the data normality test. Cross tabs with the chi-squared test were used to compare the categorical variables between the negative and positive groups. Univariable and multivariable binary logistics regression were used to test the predictive value of variables for LLN metastasis, identify independent risk factors, and establish a regression model to predict LLN metastasis. Receiver operating characteristic (ROC) curves were constructed to test the diagnostic efficacy of univariate and predictive regression models for LLN metastasis. The area under the ROC curve (AUC), Youden’s index, sensitivity, specificity, positive predictive value, and negative predictive value were calculated. Differences among AUCs were compared using the DeLong test. An AUC based on 10-fold cross-validation was generated to internally validate the prediction model.

Statistical analyses were performed using IBM SPSS (version 25; IBM Corporation Armonk, NY, United States), MedCalc software (version 15.2.2; MedCalc Software, Ostend, Belgium), and R language (version 4.0.5; R Foundation for Statistical Computing, Vienna, Austria). Statistical significance was set at P < 0.05.

## Results

### Comparison of clinicopathological and imaging features

Among the 111 eligible patients, 68 patients had LLN metastasis; the remaining 43 patients were negative for LLN metastasis without neoadjuvant therapy. The clinical characteristics and pathological and imaging features of the primary tumor and mesenteric lymph nodes were compared between patients with positive and negative LLNs (Table 1). In this sample, patients with LLN metastasis (median = 42 mm, IQR = 29–53 mm) had a shorter distance from the tumor to the anus than those without LLN metastasis (median = 57 mm, IQR = 40–70 mm, P = 0.001). The higher the clinical T stage, that is, the deeper the primary tumor invasion, the greater the likelihood of LLN metastasis in patients with rectal cancer (P = 0.010, χ^2^ = 6.565). The incidence of LLN metastasis was greater in patients with mesenteric lymph node metastasis than in patients without mesenteric lymph node metastasis on CT imaging (P = 0.003, χ^2^ = 9.004). Inter-observer agreement of EMVI was great, with a Kappa value of 0.92. EMVI-positive patients were significantly more likely to develop lymph node metastasis, which was statistically significant (P < 0.001, χ^2^ = 21.855).

Of the 140 LLNs evaluated, 76 were positive and 64 were negative for metastases, respectively. Table 2 shows the short diameter and lateral region of the target LLN, along with the status of MRA between the LLN(+) and LLN(−) groups. The consistency of MRA status between observers was relatively strong, with a Kappa value of 0.863. The MRA was more visible in the LLN(+) group than in the LLN(−) group (P < 0.001, χ^2^ = 62.153). The short diameter of the target LLN in the LLN(−) group (median = 3 mm, IQR = 2–6 mm) was smaller than that in the LLN(+) group (median = 9 mm, IQR = 7–12 mm, P < 0.001). The regional distribution of the target lymph nodes was significantly different between the two groups (P < 0.001, χ^2^ = 30.763); notably, the target lymph nodes located only in the external iliac region may be benign.

**TABLE 2:** Comparison of MRA, lymph node short diameter and lateral region between positive and negative LLN^a^ groups.

Features	Positive LLN (n = 76)	Negative LLN (n = 64)	P value
MRA^a^ status^b^			<0.001
Visible	72 (95)	20 (31)	
Non-visible	4 (5)	44 (69)	
Short diameter (mm)^c^	9 (7–12)	3 (2–6)	<0.001
Lateral region			<0.001
Internal iliac	31 (41)	12 (19)	
Obturator	33 (43)	22 (35)	
External iliac	6 (8)	29 (46)	
Multiple regions	6 (8)	0 (0)	

Note:

^a^ Abbreviations: LLN, lateral lymph node; MRA, middle rectal artery.

^b^ Categorical variable was shown as n (%).

^c^ Nonnormally distributed variables was shown as Median (interquartile range).

### Diagnostic models for predicting LLN metastasis

Univariate binary logistics regression was used to assess the odds ratio of variables for patients with metastatic LLN (Table 3) and LLN metastasis on a certain lateral side (Table 4), respectively. Multivariable logistic analysis revealed two independent risk factors for patients with positive LLN, anal distance from the tumor and EMVI (Table 3). To predict LLN metastasis on a certain lateral side, MRA status on the corresponding side, short diameter, and lateral region (external iliac alone or others) of the target lymph node were statistically significant in the univariate analysis and were also all independent risk factors in the multivariable logistic analysis (Table 4).

**TABLE 3:** Univariate and multivariable logistics regression for patients with positive LLN^a^.

Features	Univariate analysis			Multivariable analysis		
	Odds ratio	95% CI^a^	P value	Odds ratio	95% CI	P value
Anal distance (mm)	0.971	0.951, 0.990	0.003	0.962	0.938, 0.986	0.002
Clinical T stage (T1-2 vs. T3-4)	9.194	1.028, 82.239	0.047	3.503	0.337, 36.452	0.294
Mesenteric lymph node status on images (negative vs. positive)	3.052	1.201, 7.756	0.019	2.408	0.710, 8.158	0.158
EMVI^a^ on images (negative vs. positive)	8.571	2.687, 27.343	<0.001	6.610	1.752, 24.939	0.005

Note:

^a^ Abbreviations: LLN, lateral lymph node; CI, confidence interval; EMVI, extramural vascular invasion.

**TABLE 4:** Univariable and multivariable logistics regression for LLN^a^ metastasis.

Features	Univariable analysis			Multivariable analysis		
	Odds ratio	95% CI^a^	P value	Odds ratio	95% CI	P value
MRA^a^ status (Visible vs. Non-visible)	39.600	12.701, 123.467	<0.001	15.590	3.859, 62.976	<0.001
Short diameter of LLN (mm)	1.889	1.525, 2.338	<0.001	1.767	1.380, 2.263	<0.001
Lateral region (external iliac alone vs. others)	8.816	3.267, 23.792	<0.001	8.331	1.671, 41.542	0.010

^a^ Abbreviations: LLN,lateral lymph node; CI, confidence interval; MRA, middle rectal artery.

Diagnostic Model 1 for predicting LLN metastasis was constructed using the logistics regression equation with the first two independent variables (MRA status and short diameter), as follows: logit (*P*_*1−2*_) = −5.531 + 3.076 × *X1* + 0.564 × *X2*. Diagnostic Model 2 included the three independent variables: logit (*P*_*1−3*_) = −7.010 + 2.747 × *X1* + 0.569 × *X2* + 2.120 × *X3*. In each, *P* is the prediction probability of LLN metastasis, *X1* is the MRA status (non-visible = 0, visible = 1), *X2* is the short diameter of the LLN (mm), and *X3* is the lateral region (only external iliac = 0, others = 1).

### ROC analysis and comparison of diagnostic efficiency

**FIGURE 4:**
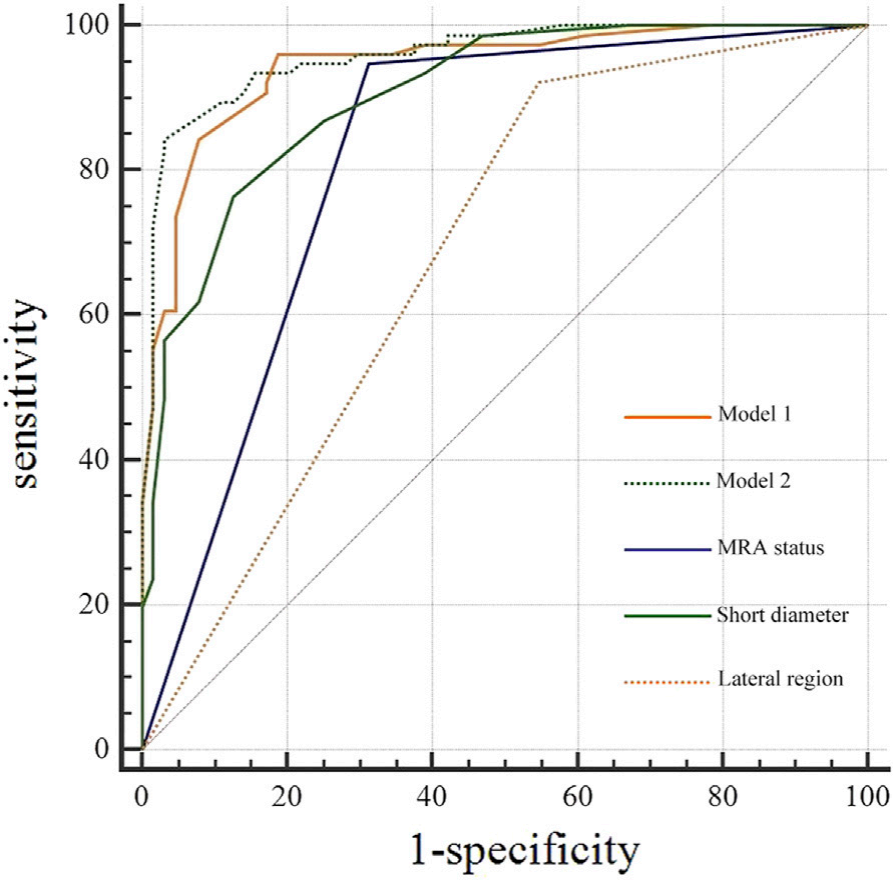
Receiver operating characteristic curve (ROC) analysis of diagnostic models, middle rectal artery (MRA) status, and short diameter for lateral lymph node (LLN) metastasis.

The diagnostic efficacy of the prediction probabilities of Models 1 and 2, as well as each single scale was evaluated using ROC analysis (Table 5; Figure 4). Both Models 1 and 2 showed superior diagnostic performance compared to the other single scales, with AUC values of 0.945 and 0.959, respectively, but there was no statistical difference (P = 0.128) between the two models themselves (Table 6). The accuracies of Models 1 and 2 were similar to one another at approximately 89.29% and 90.00%, respectively. However, Model 1 contained fewer parameters than Model 2 and may be easier to implement. For internal validation, the 10−fold cross−validation (mean AUC = 0.869, 95% confidence interval = 0.835–0.903) demonstrated a relatively reasonable performance of the Model 1.

**TABLE 5:** Diagnostic performance for LLN^a^ metastasis.

Diagnostic scales	Sen^a^ (%)	Spe^a^ (%)	PPV^a^ (%)	NPV^a^ (%)	Accuracy	AUC^a^	95% CI^a^
Model 1	96.05	81.25	85.88	94.55	89.29	0.945	0.893, 0.976
Model 2	84.21	96.87	96.97	83.78	90.00	0.959	0.911, 0.985
MRA status	94.74	68.75	78.26	91.67	82.86	0.817	0.743, 0.878
Short diameter	86.84	75.00	80.49	82.76	81.43	0.904	0.842, 0.947
Lateral region	92.11	45.31	66.67	82.86	70.71	0.687	0.603, 0.763

Note:

^a^ Abbreviation: LLN, ateral lymph node, Sen = sensitivity, Spe = specificity, PPV, positive predictive value; NPV, negative predictive value; AUC , area under the curve; CI, confidence interval.

**TABLE 6:** Comparisons of diagnostic scales using AUC^a^.

Comparisons of diagnostic scales			Difference between AUCs	P value
Model 1	vs.	Model 2	0.014	0.128
Model 1	vs.	MRA^a^ status	0.127	<0.001
Model 1	vs.	Short diameter	0.041	0.014
Model 1	vs.	Lateral region	0.258	<0.001
MRA status	vs.	Short diameter	0.086	0.025
MRA status	vs.	Lateral region	0.130	0.003
Short diameter	vs.	Lateral region	0.217	<0.001

Note:

^a^ Abbreviation: AUC, rea under the curve; MRA, middle rectal artery.

**FIGURE 5:**
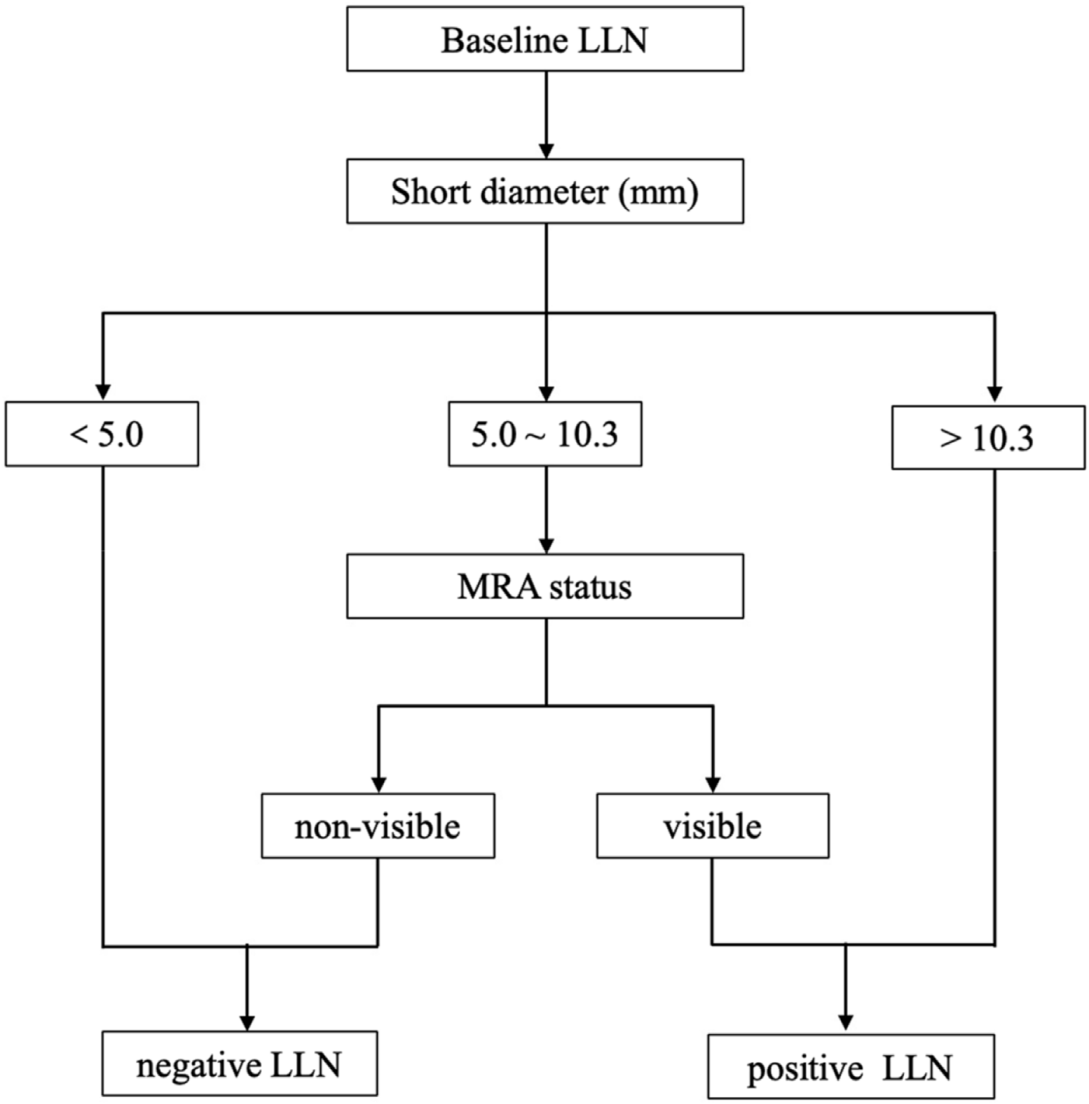
The diagnostic flow chart including short diameter of lateral lymph node (LLN) and middle rectal artery (MRA) status.

**FIGURE 6:**
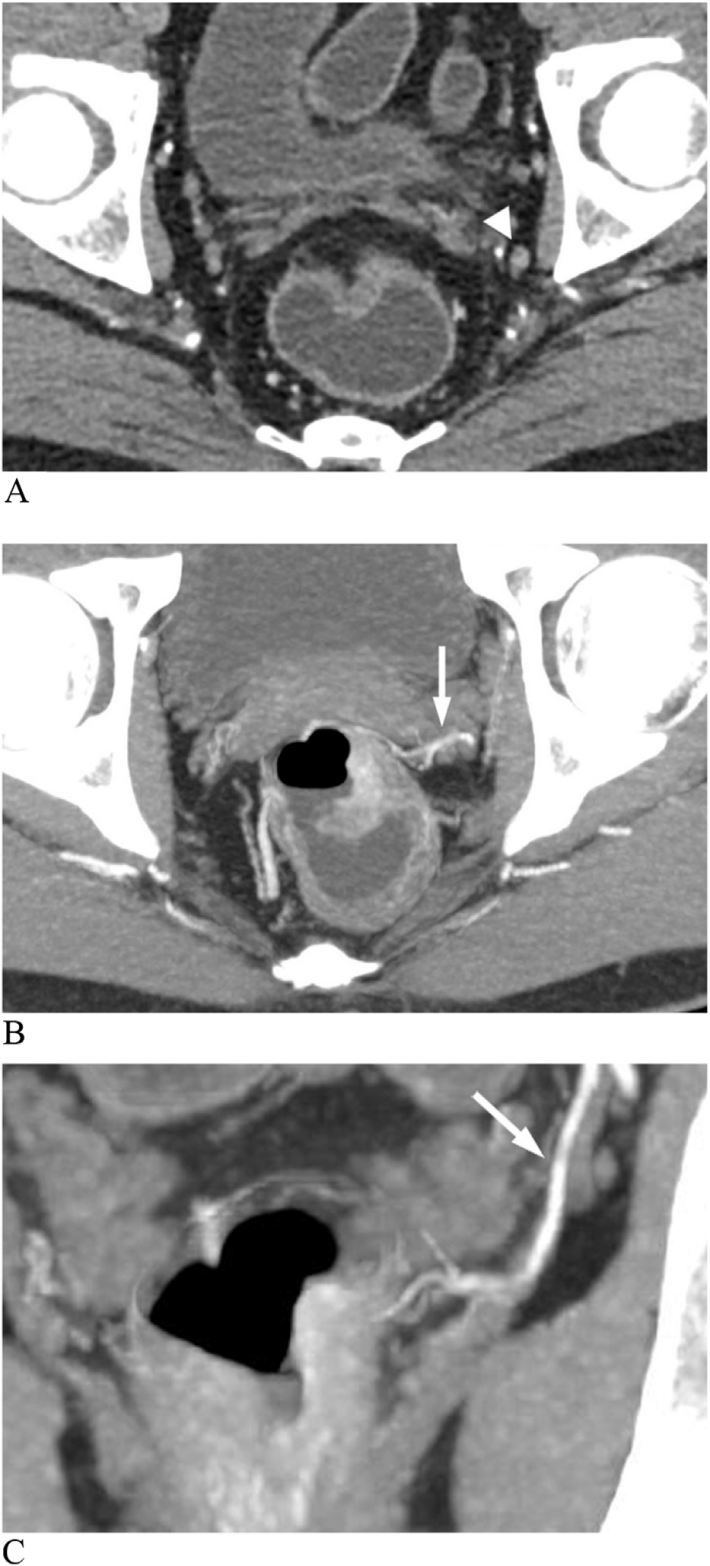
Enhanced CT images show a metastatic lateral lymph node (LLN) in rectal cancer. **(A)** Lymph node (arrowhead) with a short diameter of 5 mm. **(B)** Axial image shows an anterolateral middle rectal artery (MRA) (arrow). **(C)** Coronal image shows the left MRA (arrow) connecting the internal iliac artery to the thickened rectal wall.

If the prediction probability corresponding to Model 1 was greater than the cutoff value of 0.318, LLN metastasis was considered positive. Based on the regression equation of Model 1 and the corresponding cutoff value (0.318), a diagnostic flow chart including short diameter and MRA status was obtained (Figure 5). When the short diameter of the target LLN was <5.0 mm, Model 1 diagnosed the LLN as negative, regardless of MRA status. Conversely, when the short diameter was >10.3 mm, the LLN was considered positive, regardless of the presence of MRA. Finally, when the short diameter was between 5.0 and 10.3 mm, if the MRA was visible, then the LLN was classified as positive; otherwise, it was classified as negative (Figure 6).

For a single diagnostic scale, the sensitivity of the MRA was the highest (94.74%), whereas its specificity was moderate (68.75%). As a continuous variable, the AUC of LLN short diameter was 0.904 with a cutoff value of >6 mm, and the corresponding specificity (75.00%) was greater than that of MRA status, while the sensitivity (86.84%) was lower. The lateral region of the LLN showed poor specificity (47.27%) and high sensitivity (90.77%) (Table 5).

## Discussion

In our study, LLN metastasis was predicted using a CT-based diagnostic model combining MRA status and lymph node short diameter. This model can improve the diagnostic efficiency for LLN metastasis on the lateral side and facilitate clinical practice. As LLNs with a diameter of 5–10 mm are especially difficult to diagnose using the short-diameter criterion alone, the addition of MRA status might be helpful for accurate diagnosis.

Regardless of imaging methods, metastatic or suspicious LLNs are generally diagnosed based on the criteria of short diameter with a cutoff value of 4–12 mm (Ogawa et al., 2021; Kim et al., 2014). In our study, LLNs with short diameter of >6 mm were diagnosed as metastatic with a reasonable sensitivity and specificity values of 86.84% and 75.00%, respectively. Similar to in previous studies, 7 mm was considered the threshold for metastatic LLN (Sluckin et al., 2022; Ogura et al., 2019; Sluckin et al., 2023). In Japan, selective LLND is performed according to a size criterion of ≥7 mm, but the pathologic positive rate is not high, which is currently unsatisfactory (Akiyoshi et al., 2012; Akiyoshi et al., 2014; Christou et al., 2021). In another study, the short-diameter threshold of LLN metastasis was set at 10 mm, with high specificity (98.5%) and low sensitivity (43.8%) (Ishibe et al., 2016). However, low-sensitivity diagnostic criteria can lead to an omission of diagnosis, which means missed opportunities for timely treatment. Above all, the short-diameter criterion alone is not sufficient for the diagnosis of LLN metastasis, especially when we need a more accurate diagnosis is necessary to enable more precise treatment.

Our study showed that the MRA visualization is predisposed to LLN metastasis, and the MRA visualization is also an independent risk factor for LLN metastasis. The single MRA status scale was highly sensitive (94.74%) and moderately specific (68.75%) for diagnosing LLN metastasis. Lymphatic vessels around the MRA may provide a drainage pathway to the lateral pelvic sidewall for tumor cells (Heinze et al., 2023), while the occurrence of LLN metastasis is also affected by other factors, such as tumor location, tumor invasion depth, and EMVI (Li and Shiomi, 2021). Iwasa et al. found that MRA visualization by magnetic resonance imaging was a significant independent predictor of LLN metastasis and the MRA showed high sensitivity (95%) and low specificity (41.5%) in the diagnosis of lymph node metastasis, consistent with our results (Iwasa et al., 2021). However, our study showed the relationship between the ipsilateral MRA and lymphatic drainage more directly.

In our study, a regression model including MRA visibility and short diameter of LLN was constructed to predict lymph node status on the corresponding lateral side. The Youden index was used to find the optimal cutoff value of the diagnostic model, so as to take into account the sensitivity and specificity of lymph node metastasis diagnosis. When it is confused to identify the status of LLN, the suspicious LLN short diameter and the ipsilateral MRA status can be substituted into the model to calculate the prediction probability. If the prediction probability is greater than the cutoff value (0.318), it indicates that the suspected LLN may have metastasis. Considering the complexity of direct application of regression equations, we made a flow chart based on the equation. LLNs were considered positive when the short diameter was >10.3 mm, and negative when the short diameter was <5 mm. As reported in a previous study, a cutoff of 5 mm demonstrated a higher sensitivity of 72.6% and lower specificity of 54.7%, and meanwhile the cutoff of 10 mm had a high specificity of 96.4% but a poor sensitivity of 19.5% (Ogawa et al., 2016b). It indicated that the short-diameter criterion of LLN alone was insufficient to guide treatment, leaving some patients unable to benefit from LLND due to overtreatment or placing others at increased risk of lateral recurrence due to missed treatment opportunities. Interestingly, the application of MRA could further indicate the status of LLNs with a short diameter of 5–10.3 mm. The model combining MRA status and short diameter yielded higher sensitivity (96.05%), specificity (81.25%), and AUC (0.945) than the short-diameter criterion alone. A similar method combining two variables to predict LLN status has showed that stratification of the distance of the tumor from the anal verge, combined with lymph node size, achieved high sensitivity but low specificity (Bae et al., 2023). The rationale is that LLN metastasis of rectal cancer may occur in small nodes, and errors in size measurement are inevitable. Our diagnostic model may compensate for the insufficiency of the short−diameter criteria.

Our study has several limitations. First, biases may exist in patient selection, as those who underwent LLND usually had enlarged or visible LLN indicated by CT. Second, the target LLNs were selected based on the comparison of CT images before and after surgery, and there was no precise node-to-node correspondence between CT findings and pathology. Due to its limited sample size, this study only had internal validation and lacked external validation. Finally, our study mainly focused on the role of MRA status in the auxiliary diagnosis of LLN at baseline, and the characteristics of LLN after neoadjuvant therapy require further exploration.

This study suggests that the MRA status is associated with ipsilateral LLN metastasis. A CT-based model combining MRA status and lymph node short diameter can improve the diagnosis of LLN metastasis.

## Data Availability

The raw data supporting the conclusions of this article will be made available by the authors, without undue reservation.

## Abbreviations

MRA, middle rectal artery; LLN, lateral lymph node; CT, computed tomography; ROC, receiver operating characteristic; AUC, area under the receiver operating characteristic curve; LLND, lateral lymph node dissection; EMVI, extramural vascular invasion; IQR, interquartile range.
